# 

**Published:** 1879-02

**Authors:** 


### TABLE OF CONTENTS.

ORIGINAL COMMUNICATIONS.

Correspondence........................................................................ 641

Cure of Opium Habit. *By Wm. O'Daniel, M D., Bullards, Ga.*. 643

A Few Days' Obstetrical Experience. *By R. W. Westmoreland, M. D., Washington, Arkansas*.................................. 645

REPORTS OF SOCIETIES.

Atlanta Academy of Medicine.............................................. 647

SELECTIONS.

Alimentation in Health and Disease........................................ 655
Laparo-Elytrotomy............................................................ 669
Notes of four Cases of Leprosy in Minnesota............................ 674
Inversion of the Uterus...................................................... 680
Elementary Lessons in Electricity.......................................... 688

EDITORIAL.

Medical Associations.......................................................... 693
Atlanta Medical College...................................................... 694

BIBLIOGRAPHICAL.

Diptheria—Its Nature and Treatment........................................ 695

EXCERPTA.

Damiana as a Nerve Tonic.................................................... 696
The Application of Pressure in Treatment of Uterine Diseases........ 697
Suggestions for Sleeplessness............................................. 699
Syphilitic Milkmen............................................................ 699
German Treatment of Croup and Diptheria.................................. 700
Obstinate Vomiting Cured by Meat-Pancreas Injection.................. 701
Congenital Deformity of the Superior Extremity.......................... 701
Anæsthesia Cured by the Application of Metal............................ 702
Absence of the Radius......................................................... 703
Cysticerci in the Brain Diagnosticated during Life...................... 704

